# Factors influencing the availability of anesthesiologists: a scoping review

**DOI:** 10.1186/s12960-025-01021-3

**Published:** 2025-10-24

**Authors:** Zahra Keyvanlo, Fatemeh Kokabisaghi, Mahdi Yousefi, Alireza Sedaghat, Hossein Ebrahimipour

**Affiliations:** 1 Student Research Committee, School of Health, Mashhad University of Medical Sciences, Mashhad, Iran; 2 Non-Communicable Diseases Research Center, Sabzevar University of Medical Sciences, Sabzevar, Iran; 3 Department of Health Economics and Management Sciences, School of Health, Mashhad University of Medical Sciences, Mashhad, Iran; 4 Birmingham Centre for Evidence and Implementation Science, University of Birmingham, Birmingham, UK; 5 Lung Diseases Research Center, Mashhad University of Medical Sciences, Mashhad, Iran; 6 Social Determinants of Health Research Center, School of Health, Mashhad University of Medical Sciences, Mashhad, Iran; 7 Clinical Research Development Unit, Shahid Hasheminejad Hospital, Mashhad University of Medical Sciences, Mashhad, Iran

**Keywords:** Accessibility, Availability, Health Workforce, Anesthesia, STEEP, Anesthesiologist

## Abstract

**Introduction:**

Access to safe surgery and anesthesia remains a pressing global challenge, particularly in low- and middle-income countries (LMICs). This study examines the factors influencing the worldwide availability of anesthesiologists, a critical determinant of effective surgical care.

**Methods:**

We performed a scoping review based on the five-stage Arksey and O’Malley framework. Relevant studies were identified through systematic searches of scientific and grey literature databases, including PubMed, Scopus, Web of Science, and Google Scholar, as well as anesthesia-specific websites and journals. The review encompassed publications from May 2015 to November 2023. A multidisciplinary team conducted data extraction and thematic coding using the STEEP(Social, Technological, Economic, Environmental, Political) model, resolving discrepancies through consensus.

**Results:**

Of the 925 screened articles, 63 met the inclusion criteria. The analysis identified 68 distinct factors organized into five STEEP areas. The most frequently cited issues in each category are as follows: (1) Political Factors: These emerged as the most frequently cited, with national and international institutional support for workforce planning identified as a cornerstone issue. (2) Social Factors: Key challenges included limited opportunities for professional development and skills evaluation, compounded by poor work-life balance. (3) Economic Factors: Financial disincentives and excessive workloads stood out as the primary barriers to anesthesiologist availability. (4) Technological Factors: Restricted access to medical training opportunities posed a significant obstacle. (5) Environmental Factors: Though less prominent, these were recognized for their potential to enhance geographical equity and resource access in anesthesia education and service delivery. High-income countries focused on optimizing performance, improving workplace quality, and strengthening retention strategies, while LMICs contended with structural challenges such as resource shortages, workforce migration, and inadequate infrastructure. Across all countries, social issues such as job burnout and work-life imbalance, alongside economic challenges like financial incentives and workload were recurring themes.

**Conclusions:**

These findings illuminate the complex, multifaceted nature of factors affecting anesthesiologist availability. They underscore the necessity for comprehensive strategies that promote collaboration at local, national, and global levels. Addressing the political, economic, social, technological, and environmental dimensions is imperative to ensure safe and effective anesthesia care worldwide.

**Supplementary Information:**

The online version contains supplementary material available at 10.1186/s12960-025-01021-3.

## Introduction

Access to safe anesthesia services is vital to curative services, especially in operative rooms and urgent surgeries. Despite the World Health Organization's(WHO) emphasis on universal health coverage, the shortage and inequitable distribution of anesthesiologists, especially in low- and middle-income countries, are among the major obstacles to achieving this global goal (1). Anesthetists play a central role in the safe management of anesthesia, interdisciplinary collaboration, and improving surgical outcomes (2). An anesthetist is considered the first line of medical care providers during epidemics and engages in extensive cooperation with other medical specialties, especially surgeons (3, 4). Due to the shortage of anesthesiologists in some countries, nurses provide anesthesia services, which greatly impacts the costs of treatment and patient safety. The presence of an anesthesiologist in the surgical team can improve surgical outcomes. They are anesthesia care team leaders and supervise the activities of non-physician anesthesia providers (5, 6).

Strengthening essential surgical care and anesthesia as part of universal health coverage efforts was emphasized by the World Health Assembly in 2015. For this purpose, the Lancet World Surgical Commission recommended six leading indicators for all governments to monitor the progress of this type of care. One indicator is related to the number of available surgeons, anesthesiologists, and obstetricians (SAO) per 100,000 populations. To have fair access, at least 20 surgeons, anesthesiologists, and obstetricians are needed for every 100,000 people (7, 8). However, one of the main challenges in safe anesthesia management is the shortage of anesthesiologists. Reports indicate that five billion people worldwide face challenges to access anesthesia care, with more severity in low- and middle-income countries. Moreover, 136,000 additional anesthesiologists are needed globally to achieve the Universal Health Coverage index (7, 8). With proper planning and distribution of specialized human resources, the availability of health care can be improved (9, 10). Although the indicator of the number of SAOs available per 100,000 people is very important for human resources planning, it cannot express the complexities and challenges of different health systems. The provision of anesthesia services in low- and middle-income countries is influenced by factors such as access to health infrastructure, political stability, economic and cultural conditions, and skilled labor. Factors such as fair rewards and financial incentives, autonomy of anesthesiologists, access to educational resources, and job burnout affect the capacity of the anesthesia workforce (11, 12). Therefore, a detailed and realistic analysis of barriers to the availability of anesthesiologists should be considered more than demographic indicators. To increase the availability of anesthesia care, it is necessary to identify political, economic, environmental, social, and technological factors and to plan effectively (13–15). This study was conducted to identify the factors affecting the availability of anesthesiologists worldwide.

## Materials and methods

In this study, we conducted a scoping review. This type of review aims to outline the fundamental concepts of a research area and identify the types of evidence available (16, 17). We prepared the study protocol based on the Arksey and O'Malley framework (18, 19). The protocol includes six steps:(a) defining the research question,(b) identifying studies by the search strategy,(c) selecting studies,(d) extracting the data,(e) collecting, summarizing, analyzing, and reporting the results, and(f) consulting with stakeholders, which is optional. Considering that the research team consisted of human resources planning and anesthesiology experts, their opinions were used to confirm the results. The details are provided as follows.

### Definition of the research question

What factors have been reported in improving anesthesia workforce access following the 2015 global policy shift (Adoption of resolution World Health Assembly Resolution 68.15 on Strengthening Emergency and Essential Surgical Care and Anesthesia as a Component of Universal Health Coverage)?

### Identification of studies

Three research team members (Z.K., H.E, and F.K.) determined the inclusion criteria by reviewing the references of related studies. These studies addressed the availability, enhancers, barriers, and challenges of anesthesiologist access following the 2015 global policy shift. Subsequently, a university librarian and the research team developed the search strategy.

#### Inclusion criteria

Medical Subject Heading (MESH) keywords, including supply and distribution, workforce, health services accessibility, and anesthesiologist, were used to search PubMed and as a guide for other databases. Original articles, reviews, editorials, and reflective articles, including expert opinions on the topic, were included (20–22). We found related studies by searching scientific databases such as PubMed, Scopus, Web of Science, Google Scholar, and grey literature in anesthesia-related websites. Papers published between May 2015 and November 2023 were included in the study. May 2015 was the adoption date of the World Health Assembly Resolution 68.15 on “Strengthening Emergency and Essential Surgical Care and Anesthesia as a Component of Universal Health Coverage” (23). The selection of studies published from May 2015 onward was a deliberate and methodologically grounded decision, reflecting the core objective of this review. Although it is well recognized that anesthesia workforce shortages are not a recent phenomenon, the adoption of World Health Assembly Resolution 68.15 in May 2015 represented a significant policy inflection point. This resolution repositioned anesthesia and surgical workforce development as priorities within the global health policy discourse.

In the aftermath of WHA 68.15, a substantial body of scholarly and policy-oriented work emerged, characterized by the development of national surgical plans, strategic workforce assessments, and expanded international reporting mechanisms. Notably, many of these efforts explicitly examined the structural determinants of workforce availability, service delivery constraints, and enabling contextual factors—particularly in low- and middle-income countries (LMICs). Restricting this review to the post-2015 literature enabled a focused analysis of studies that not only acknowledged the longstanding nature of the anesthesia workforce crisis, but also evaluated it through the lens of emerging patterns, system-level adaptations, and policy-driven innovations that have taken shape in direct response to this global mandate. These studies tend to offer actionable strategies, evaluative frameworks, and implementation insights—critical components for informing future-oriented decisions in health workforce planning (24).

#### The keywords used in the search strategy included:

Anesthesiologist, Physician Anesthetist, Anesthesiology Workforce, Physician anesthesia providers, Anesthesia Workforce, Anesthesia Services, Access, Availability, Geographic Accessibility, Geographic Distribution, Supply, Demand, Distribution, Workforce Planning, Health Workforce, Human Resources, Staffing, Need.

Population: Studies addressing the issue of access to anesthesiologists following the global policy change in 2015.

Concept: Studies that identify factors affecting access, barriers, facilitators, or challenges following the global policy change in 2015 and that directly address at least one of the following topics:

- Trends affecting the supply and distribution of anesthesiologists
• Challenges and barriers to access to anesthesia services
• Human resource planning in anesthesia
• Experiences and perspectives of professionals or policymakers on the anesthesia workforce

Context: Articles published between May 2015 and November 2023 in any country at any income level (these articles must be fully accessible and published in English).

#### Exclusion criteria

The exclusion criteria were conference abstracts, duplicates, unavailable full-text papers, and papers in a non-English language, papers addressing non-physician anesthesia service providers, and articles that presented only statistics and did not have analysis.

### Selection of studies

In the initial search, 889 studies were extracted and entered into Endnote version 20 software. After removing the duplicates by software and manually (277 studies), 612 unique citations remained. The titles and abstracts of the studies based on pre-defined inclusion and exclusion criteria were screened (160 studies). Two of our study team members (Z.K. and F.K.) reviewed the papers. If there was a problem regarding the study selection, we consulted the third reviewer (H.E.).

**Fig. 1:**
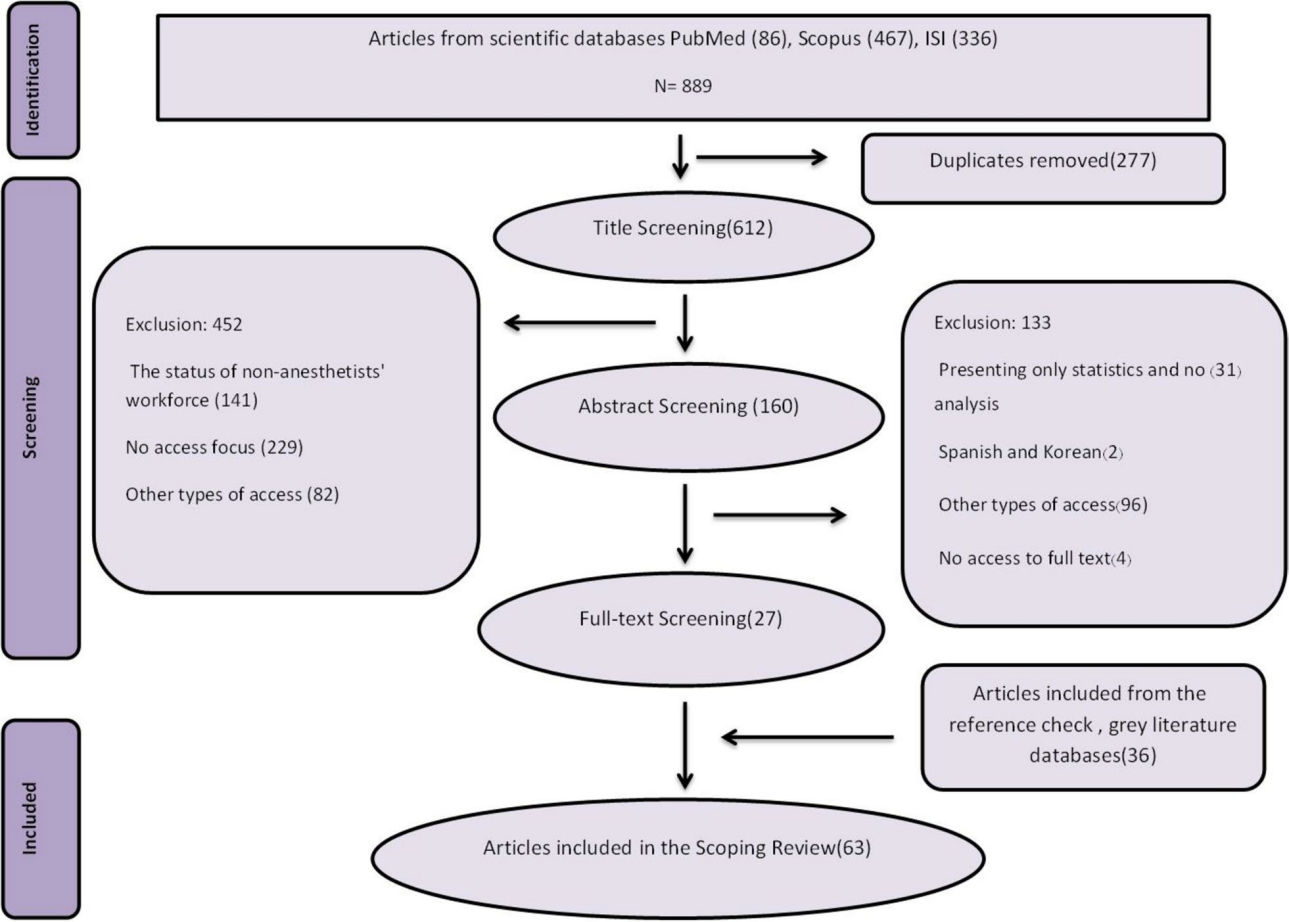
PRISMA Diagram

In the next step, the full texts of 160 studies were screened. At this level, 133 articles were excluded due to reasons (see Fig. 1 for exclusion reasons). A total of 27 articles met the inclusion criteria. To enhance comprehensiveness, in addition to database searches, we conducted a targeted manual search of grey literature sources. These included the official website of the Royal College of Anaesthetists (RCoA, Churchill House, London) and the official websites of high-impact anesthesiology journals, including Anesthesiology, Anaesthesia, British Journal of Anaesthesia (BJA), Anesthesia & Analgesia, and Canadian Journal of Anesthesia (CJA), to identify relevant editorials, Guidelines, and surveys not indexed in Scientific databases. This yielded an additional 36 studies. We included 63 studies in the final stage. The full study selection process, including records identified, screened, excluded, and included, is detailed in Fig. 1, according to PRISMA guidelines.

### Data extraction

We used Microsoft Excel to summarize the extracted articles. Data were independently extracted by two reviewers (Z.K. and F.K.) for all included studies. Discrepancies were resolved through discussion or adjudication by a third reviewer (H.E.). After reaching a consensus on the final articles, two members of the research team (Z.K. and F.K.) extracted and summarized the information of all the studies, including the names of the authors, the year of publication, the type of study, the studied country, and factors affecting the availability of anesthesiologists (Table 1 of the Supplementary File).

**Table 1:** Characteristics of the reviewed studies

	Author (Year)	Type of Study	Country income level	Factors affecting the availability of anesthesiologists
1	Cooper, M. G.(2020)(58)	Editorials	Kenya	Equitable geographic distribution of resources and physicians
2	Methangkool,E.(2023)(92)	Review	US	Gender Differences
3	Mumbwe, M.C(2020)(32)	cross-sectional	Zambia	Job burnout, access to facilities, equipment, medicine, professional relationships, communication, clinical independence, recognition/referral by other colleagues, especially surgical staff, and agreement between clinical positions in intraoperative decisions and working hours
4	Basile, E. R.(2023)(33)	Qualitative	International	Gender Differences
5	Li, H.(2018)(97)	cross-sectional	China	Job burnout, the age and gender of the specials, workload, the characteristics of hospitals at referral levels
6	Lundeg, G.(2018)(75)	Review	Mongolia	The length of the training period, workload, medical training opportunities, immigration, the support of national and international organizations, access to facilities, equipment, the time spent between the places of residence and work (urban/rural),Equitable geographic distribution of resources and physicians
7	Shaw, A. D.(2023) (66)	Editorial	US	Financial incentives, workload, Medical training opportunities
8	Enright, A. (2017)(48)	Editorial	Canada	Medical training opportunities, the support of national and international organizations, professional relationships, communication, clinical independence, recognition/referral by other colleagues, especially surgical staff, and agreement between clinical positions in intraoperative decisions and working hours, distance education
9	Drum, E. T.(2017)(37)	Editorial	Canada	The support of national and international organizations, Task Sharing, medical and technological advances, distance education
10	Emala, C.W. S.(2023)(72)	Review	US	The support of national and international organizations, financial incentives, medical training opportunities, adequate opportunities for professional development and assessment of competence, job characteristics, workload, the length of the training period, The support of the hospital manager or policymakers
11	Nwokolo, O. O.(2022)(70)	Review	US	Job burnout ،medical training opportunities ،, adequate opportunities for professional development and assessment of competence, Gender Differences, the age and gender of the specialist, financial incentives
12	Shaefi, S.(2023)(49)	Survey	US	Job characteristics, workload, job burnout, professional relationships, communication, clinical independence, recognition/referral by other colleagues, especially surgical staff, and agreement between Clinical positions in intraoperative decisions and working hours, incompatibility of personal life with work, medical training opportunities, access to facilities, equipment, medicine
13	Meadows, J.W.(2020)(50)	cross-sectional	Bangladesh	Equitable geographic distribution of resources and physicians, access to facilities, equipment, medicine, medical training opportunities, international collaboration on two-way learning about disease patterns, treatment algorithms, research and standards of care, The number of graduations
14	Hertzberg, L..(2021)(73)	cross-sectional	US	Gender differences
15	Khan, F. A. (2022)(100)	cross-sectional	Pakistan	Equitable geographic distribution of resources and physicians, medical training opportunities, adequate opportunities for professional development and assessment of competence, planning based on local needs and resources, medical and technological advances, financial incentives
16	Orser, B. A. (2019)(34)	Review	International	Equitable geographic distribution of resources and physicians, access to facilities, equipment, medicine, financial incentives, adequate opportunities for professional development and assessment of competence, Task Sharing, medical and technological advances,, workload, distance education, medical training opportunities, the support of national and international organizations, international collaboration on two-way learning about disease patterns, treatment algorithms, research and standards of care, job burnout, the time spent between the places of residence and work (urban/rural)
17	Asingei, J. (2023)(74)	cross-sectional	East, Central, and Southern Africa	Equitable geographic distribution of resources and physicians, immigration, workload,medical training opportunities, the support of national and international organizations, adequate opportunities for professional development and assessment of competence, access to facilities, equipment, medicine, financial incentives
18	Mayes, L. M.(2018)(67)	cross-sectional	US	Gender Differences, the age and gender of the specialists
19	Kudsk-Iversen, S. (2018)(11)	Review	International	The use of internationally educated physicians, stability and political freedom, job security, the support of the hospital manager or policymakers, the equitable geographic distribution of resources and physicians, the support of national and international organizations, Task Sharing, immigration and brain drain, maternity leave, disease burden or epidemics, age and gender characteristics of the population, job characteristics, adequate opportunities for professional development and assessment of competence, incompatibility of personal life with work, employment and job opportunities, financial incentives, workload, job burnout, access to medical training opportunities, access to facilities, equipment, medicine, job satisfaction, the economic status of the countries, distance education, international collaboration on two-way learning about disease patterns, treatment algorithms, research and standards of care, medical and technological advances, robust information systems for planning
20	Khan, I.A. (2023)(1)	Editorial	International	The support of the hospital manager or policymakers, equitable geographic distribution of resources and physicians, the support of national and international organizations, Task Sharing, demand for medical, surgical and acute care, job characteristics, adequate opportunities for professional development and assessment of competence, financial incentives, workload, alternative professional activities and willingness to work in the private sector, job burnout, access to medical training opportunities, job satisfaction, the economic status of the countries, tuition fees for educational courses
21	Davies, J.I. (2018)(23)	cross-sectional	International	The number of graduations, Task Sharing, familiarity with the field, and attraction to the field
22	El Vilaly, M.A. (2021)(105)	cross-sectional	Nigeria	University acceptance rate, equitable geographic distribution of resources and physicians, population growth or life expectancy, age and gender characteristics of the population, access to city facilities, incompatibility of personal life with work, financial incentives, workload, access to facilities, equipment, medicine, robust information systems for planning, road facilities
23	Haller, G. (2021)(47)	cross-sectional	Switzerland	University acceptance rate, the number of graduations, the use of internationally educated physicians, the length of the training period, employment types, Job requirements(Standard and pattern of activity), the characteristics of hospitals at referral levels, equitable geographic distribution of resources and physicians, protocols, administrative responsibility of specialists, Task Sharing, immigration and brain Drain, the age and gender of the specialist, gender differences, the rate of retirement and death, maternity leave, disease burden or epidemics, population growth or life expectancy, age and gender characteristics of the population, demand for medical, surgical and acute care, familiarity with the field, adequate opportunities for professional development and assessment of competence, incompatibility of personal life with work, financial incentives, workload, alternative professional activities and willingness to work in the private sector, access to medical training opportunities, attraction with the field, access to facilities, equipment, medicine, medical and technological advance, the variety of anesthesia services
24	Department of Health and Aged Care (2015)(35)	Report	Australia	University acceptance rate, the length of the training period, job requirements(Standard and pattern of activity), equitable geographic distribution of resources and physicians, Task Sharing, the age and gender of the specialist, gender differences, maternity leave, population growth or life expectancy, age and gender characteristics of the population, demand for medical, surgical and acute care, leaving or changing jobs, incompatibility of personal life with work, employment and job opportunities, financial incentives, access to medical training opportunities, access to facilities, equipment, medicine
25	Muffly, M.K.(2018)(59)	Longitudinal Analysis	The U.S	The number of graduations, job requirements(Standard and pattern of activity), the equitable geographic distribution of resources and physicians, the age and gender of the specialist, the rate of retirement and death, population growth or life expectancy, demand for medical, surgical and acute care, job characteristics, familiarity with the field, employment and job opportunities, alternative professional activities and willingness to work in the private sector, attraction with the field, tuition fees for educational courses
26	Zhou, Y.2021)(60)	cross-sectional	The U.S	The number of graduations, employment types, the characteristics of hospitals at referral levels, the equitable geographic distribution of resources and physicians, regulatory mechanisms and licensing, regulations, how to financing, the administrative responsibility of specialists, Task Sharing, the age and gender of the specialist, gender differences, the rate of retirement and death, maternity leave, age and gender characteristics of the population, demand for medical, surgical and acute care, access city facilities Economic area: employment and job opportunities, financial incentives, access to facilities, equipment, medicine, the share of the health system budget from the gross national product, the economic status of the countries, robust information systems for planning
27	Muffly, M.K.(2016)(61)	cross-sectional	The U.S	The number of graduations, employment types, the equitable geographic distribution of resources and physicians, obligations after the training period, the age and gender of the specialist, the rate of retirement and death, maternity leave, incompatibility of personal life with the workplace
28	Meara, J.G.(2015)(24)	Review	International	The number of graduations, planning based on local needs and resources, activity in two places, equitable geographic distribution of resources and physicians, regulatory mechanisms and licensing, protocols, the support of national and international organizations, Task Sharing, change of disease pattern, immigration and brain drain, population growth or life expectancy, age and gender characteristics of the population, professional relationships, communication, clinical independence, recognition/referral by other colleagues, especially surgical staff, and agreement between clinical positions in intraoperative decisions and working hours, familiarity with the field, adequate opportunities for professional development and assessment of competence, leaving or changing jobs, workload, attraction with the field, access to facilities, equipment, medicine, international collaboration on two-way learning about disease patterns, treatment algorithms, research and standards of care, medical and technological advances, robust information systems for planning, the time spent between the places of residence and work (urban/rural), Sustainable ecosystem, tuition fees for educational courses
29	Cooper, M.G.(2016)(62)	Editorial	the Pacific region	The number of graduations, planning based on local needs and resources, Task Sharing, the rate of retirement and death, age and gender characteristics of the population, professional relationships, communication, clinical independence, recognition/referral by other colleagues, especially surgical staff, and agreement between clinical positions in intraoperative decisions and working hours, job characteristics, workload, job satisfaction
30	Simkin, S. (2023)(76)	cross-sectional	Canada	The number of graduations, job requirements (Standard and pattern of activity), equitable geographic distribution of resources and physicians, immigration, the age and gender of the specialist, the rate of retirement and death, leaving or changing jobs, incompatibility of personal life with work, workload
31	Orser, B.A. (2020)(77)	Review	Canada	The use of internationally educated physicians, job requirements(Standard and pattern of activity), planning based on local needs and resources, regulatory mechanisms and licensing, social accountability, the age and gender of the specialist, maternity leave, job characteristics, access to city facilities, adequate opportunities for professional development and assessment of competence, incompatibility of personal life with work, financial incentives, distance education
32	Simkin, S.(2023)(68)	Longitudinal Analysis	Canada	The use of internationally educated physicians, the equitable geographic distribution of resources and physicians, the age and gender of the specialist, the rate of retirement and death, disease burden or epidemics, population growth or life expectancy, demand for medical, surgical and acute care, leaving or changing jobs, financial incentives, workload, job burnout, robust information systems for planning, the variety of anesthesia services, place of medical education
33	Ulisubisya, M. (2016)(63)	Review	Tanzania	The use of internationally educated physicians, the time spent between the places of residence and work (urban/rural)
34	Chan, D.M. (2016)(78)	Mixed methods	Rwanda	The use of internationally educated physicians, Task Sharing, maternity leave, incompatibility of personal life with work, employment and job opportunities, financial incentives, workload, access to facilities, equipment, medicine, and job burnout
35	Lyon, C.B.(2016)(51)	Qualitative	Mozambique	The length of the training period, activity in two places, equitable geographic distribution of resources and physicians, obligations after the training period, Task Sharing, maternity leave, familiarity with the field, adequate opportunities for professional development and assessment of competence, incompatibility of personal life with work, financial incentives, attraction with the field, access to facilities, equipment, medicine, international collaboration on two-way learning about disease patterns, treatment algorithms, research and standards of care, the time spent between the places of residence and work (urban/rural), tuition fees for educational courses
36	Khuwaja, A.(2023)(69)	cross-sectional	Pakistan	Employment types, the characteristics of hospitals at referral levels, the equitable geographic distribution of resources and physicians, Task Sharing, workload, job burnout, and robust information systems for planning
37	Hewitt-Smith, A. (2018)(89)	Mixed methods	Uganda	Employment types, brain drain, professional relationships, communication, clinical independence, recognition/referral by other colleagues, especially surgical staff, agreement between clinical positions in intraoperative decisions and working hours, job characteristics, familiarity with the field, adequate opportunities for professional development and assessment of competence, financial incentives, workload, attraction with the field
38	Carey, C.(2018)(45)	Review	International	Job requirements(Standard and pattern of activity), regulations, Task Sharing, immigration and brain drain, the age and gender of the specialist, the rate of retirement and death, disease burden or epidemics, age and gender characteristics of the population, demand for medical, surgical and acute care, employment and job opportunities medical and technological advances, the variety of anesthesia services
39	Davies, M.(2022)(83)	Guidelines	International	Planning based on local needs and resources, regulations, maternity leave, disease burden or epidemics, community health status, job characteristics, incompatibility of personal life with work, financial incentives, workload, job burnout, and job satisfaction
40	Yang, L.(2017)(52)	Survey	China	Planning based on local needs and resources, activity in two places, professional relationships, communication, clinical independence, recognition/referral by other colleagues, especially surgical staff, an agreement between clinical positions in intraoperative decisions and working hours, adequate opportunities for professional development and assessment of competence, workload, the economic status of the countries
41	Law, T.(2019)(86)	Review	International	Planning based on local needs and resources, regulatory mechanisms and licensing, adequate opportunities for professional development and assessment of competence, employment and job opportunities, workload, access to facilities, equipment, medicine, distance education, and robust information systems for planning
42	Epiu, I. (2017)(86)	cross-sectional	East Africa	Planning based on local needs and resources, equitable geographic distribution of resources and physicians, protocols, Task Sharing, familiarity with the field, financial incentives, workload, alternative professional activities and willingness to work in the private sector, attraction with the field, access to facilities, equipment, medicine, the share of the health system budget from the gross national product, efficient referral systems
43	Brouillette, M.A. (2017)(71)	Observational	Ghana	Stability and political freedom, immigration, job characteristics, familiarity with the field, financial incentives, and attraction to the field
44	Khan, F.A. (2018)(94)	Review	International	Job security, the support of the hospital manager or policymakers, the support of national and international organizations, Task Sharing, Immigration and brain drain, women's empowerment, population growth or life expectancy, demand for medical, surgical and acute care, familiarity with the field, adequate opportunities for professional development and assessment of competence, security and social justice, employment and job opportunities, attraction with the field, financial incentives, access to facilities, equipment, medicine, the share of the health system budget from the gross national product, distance education, sustainable ecosystem, tuition fees for educational courses
45	Hinkelmann, J.(2018)(64)	Review	German	The characteristics of hospitals at referral levels, the age and gender of the specialist, gender differences, maternity leave, demand for medical, surgical and acute care, professional relationships, communication, clinical independence, recognition/referral by other colleagues, especially surgical staff, and agreement between clinical positions in intraoperative decisions and working hours, Incompatibility of personal life with work
46	Rama-Maceiras, P. (2015)(43)	Review	International	The support of the hospital manager or policymakers, maternity leave, professional relationships, communication, clinical independence, recognition/referral by other colleagues, especially surgical staff, and agreement between clinical positions in intraoperative decisions and working hours, job characteristics, leaving or changing jobs, incompatibility of personal life with work, job burnout
47	Dohlman, L.E. (2017)(53)	Review	International	The support of the hospital manager or policymakers, equitable geographic distribution of resources and physicians, regulations, the support of national and international organizations, how to financing, Task Sharing, change of disease pattern, immigration, gender differences, maternity leave, community health status, professional relationships, communication, clinical independence, recognition/referral by other colleagues, especially surgical staff, and agreement between clinical positions in intraoperative decisions and working hours, job characteristics, familiarity with the field, adequate opportunities for professional development and assessment of competence, incompatibility of personal life with work, employment and job opportunities, financial incentives, attraction with the field, access to facilities, equipment, medicine, job satisfaction, War and sanctions, tuition fees for educational courses
48	Sousa, A.R.C. (2018)(54)	literature review	International	The support of the hospital manager or policymakers, equitable geographic distribution of resources and physicians, legal issues and complaints, the administrative responsibility of specialists, immigration, gender differences, the rate of retirement and death, demand for medical, surgical and acute care, professional relationships, communication, clinical independence, recognition/referral by other colleagues, especially surgical staff, and agreement between clinical positions in intraoperative decisions and working hours, job characteristics, adequate opportunities for professional development and assessment of competence, incompatibility of personal life with work, security and social justice, financial incentives, workload, job burnout, the economic status of the countries
49	Fernandes, N.L. (2023)(55)	Review	International	Equitable geographic distribution of resources and physicians, Task Sharing, immigration, financial incentives, and access to facilities, equipment, and medicine
50	Merchant, A.(2015)(38)	Survey	Guatemala, Guyana, Laos, Mozambique	Equitable geographic distribution of resources and physicians, Task Sharing, and access to facilities, equipment, and medicine
51	Zhang, C.(2021)(79)	cross-sectional	China	Equitable geographic distribution of resources and physicians, familiarity with the field, employment and job opportunities, financial incentives, workload, job burnout, attraction with the field, access to facilities, equipment, medicine, and job satisfaction
52	The Royal College of Anaesthetists: Churchill House (2020)(80)	Report	U.K	Equitable geographic distribution of resources and physicians, regulations, taxes, the age and gender of the specialist, demand for medical, surgical and acute care, employment and job opportunities, financial incentives, the economic status of the countries, and medical and technological advances
53	Zha, Y.T. (2021)(84)	cross-sectional	Guatemala	Protocols, the rate of retirement and death, robust information systems for planning
54	Wang, J.-O.(2015)(90)	cross-sectional	Taiwan	Duty system, insurance laws, maternity leave, professional relationships, communication, clinical independence, recognition/referral by other colleagues, especially surgical staff, agreement between clinical positions in intraoperative decisions and working hours, incompatibility of personal life with work, financial incentives, workload, and job satisfaction
55	Baron, E.L.(2020)(93)	Review	International	The rate of retirement and death, maternity leave, professional relationships, communication, clinical independence, recognition/referral by other colleagues, especially surgical staff, an agreement between clinical positions in intraoperative decisions and working hours, adequate opportunities for professional development and assessment of competence, leaving or changing jobs, incompatibility of personal life with work, job burnout
56	Gajewski, J.(2020)(56)	Mixed methods	Malawi, Tanzania, and Zambia	Task Sharing, social accountability, maternity leave, employment and job opportunities, financial incentives, workload, job burnout
57	Baird, M.(2015)(85)	Survey	The U.S	Task Sharing, the age and gender of the specialist, gender differences, the rate of retirement and death, age and gender characteristics of the population, demand for medical, surgical and acute care, and the economic status of the countries
58	Baxter, L.S (2017)(65)	Observational	Madagascar	Access city facilities, access to facilities, equipment, medicine, Task Sharing
59	Bouchard, M.E. (2020)(81)	Cross-sectional	Uganda, Sierra Leone	Financial incentives, workload, Task Sharing, robust information systems for planning
60	Federspiel, F. (2015)(91)	Mixed methods	International	Task Sharing, international collaboration on two-way learning about disease patterns, treatment algorithms, research and standards of care
61	Skelton, T(2020) (40)	Qualitative	Rwanda	The time spent between the places of residence and work (urban/rural), workload, job requirements(Standard and pattern of activity), medical training opportunities, the support of national and international organizations, Job satisfaction, familiarity with the field, adequate opportunities for professional development and assessment of competence, Social accountability, immigration, brain drain, leaving or changing jobs, financial incentives, professional relationships, communication, clinical independence, recognition/referral by other colleagues, especially surgical staff, and agreement between clinical positions in intraoperative decisions and working hours, activity in two places, the support of the hospital manager or policymakers, incompatibility of personal life with work, access to facilities, equipment, medicine, job burnout, attraction the field
62	Law, T. (2020)(12)	Survey	Uganda	Job satisfaction, the time spent between the places of residence and work (urban/rural), social accountability, job characteristics, medical training opportunities, access to city facilities, adequate opportunities for professional development and assessment of competence, financial incentives, workload, incompatibility of personal life with work, access to facilities, equipment, medicine, activity in two places, alternative professional activities and willingness to work in the private sector
63	Cooper, A. E.(2017)(57)	Review	U.K	Demand for medical, surgical, and acute care, job burnout, workload, equitable geographic distribution of resources and physicians, international collaboration, medical and technological advances, task Sharing, adequate opportunities for professional development and assessment of competence, support of national and international organizations, professional relationships, financial incentives, medical training opportunities, security and social justice

### Collecting, summarizing, analyzing, and reporting the results

We synthesized the findings through an analytical framework designed to map the multidimensional factors influencing anesthesiologist workforce availability. Specifically, we applied the STEEP framework, which categorizes external determinants into five domains: social, technological, economic, environmental, and political. This framework analysis is more comprehensive than other external environment analysis models and is used as a basic model in future research. This framework was selected for its broad applicability in strategic health workforce planning and its utility in future-oriented analyses (25–30). Data extracted from the included studies were charted and thematically organized under the STEEP domains. Three reviewers (Z.K., F.K., and M.Y.) with expertise in health workforce planning independently classified the data. Discrepancies in categorization were resolved through consensus discussions with a fourth expert (H.EP.), who specializes in health system future research, and a consultant anesthesiologist (A.S).

**Fig. 2:**
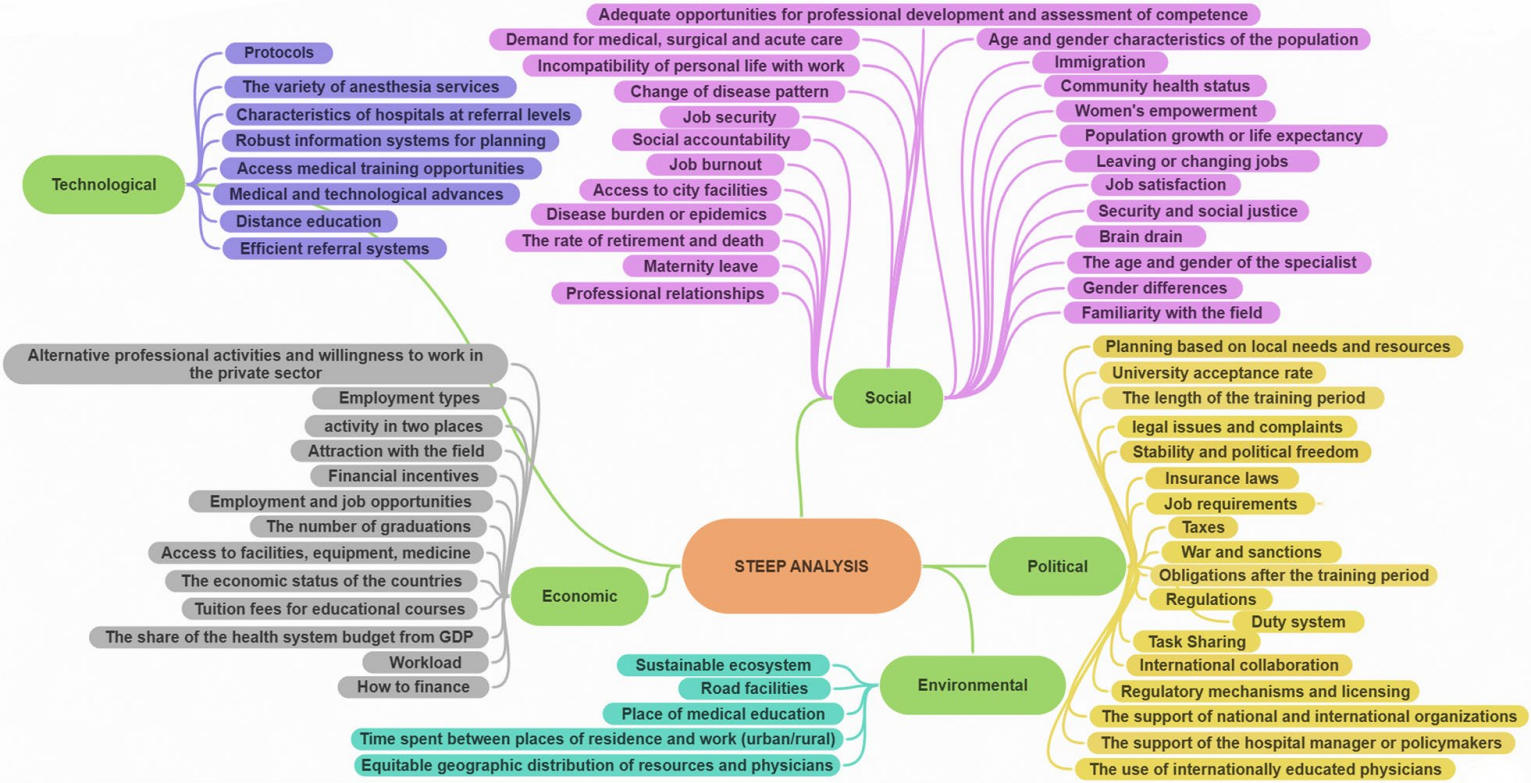
Categories of Factors Influencing the Availability of Anesthesiologists using STEEP Analysis

The factors affecting the availability of the anesthesiologist workforce are presented in Fig. 2 and Table 1 of the Supplementary File. It presents a coherent analytical framework capturing the external pressures, enabling conditions, and structural challenges that influence the strategic planning and distribution of the anesthesia workforce in diverse economic contexts.

## Results

### Characteristics of the included studies

Among the selected studies, 61 were published in scientific journals, and two were reports of the Royal College of Anesthetists and the Department of Health and Aged Care. Concerning the type of paper, three were qualitative studies, 28 descriptive-analytical studies, six editorials, four mixed methods, one guideline, two reports, and 19 reviews (Table 1 of the Supplementary File). Most studies were conducted in 2018 and 2023 (11 studies each), and the fewest were conducted in 2019 and 2022. Fifteen studies examined the state of the anesthesia workforce internationally without focusing on a specific country. Moreover, 25 studies focused on medium- to high-income countries and 21 on low- to medium-income countries. Some of the included studies used regional groupings such as 'East Africa,' 'Sub-Saharan Africa,' 'Pacific region,' or 'International.' These categories do not align directly with official income-level classifications (e.g., World Bank classification), as they often encompass countries with diverse economic statuses. Therefore, direct attribution of income level to these groupings was not possible. Table 1 of the Supplementary File outlines the characteristics of the review studies.

### Qualitative analysis results

We identified 68 factors in five political, social, economic, technological, and environmental areas. Most factors were related to the political area, and the fewest were related to the environmental area (Fig. 2). Identified factors are presented separately in Table 1 of the Supplementary File.

### STEEP analysis components

#### Political area

The political area relates to external factors such as government, trade, and tax policies, general political issues, changes in leadership, regulations, and political trends that need to be considered when evaluating the attractiveness of a labor market. Eighteen factors were identified in the political area. The most common factor was the support of national and international organizations. The least mentioned were the duty system, taxes, legal issues and complaints, War and sanctions, and insurance laws. Task Sharing has been proposed as a common solution in all countries to deal with the shortage of human resources in the field of anesthesia. Job requirements (standard and pattern of activity) have been more important in high-income countries. Support of national and international organizations and planning based on local needs and resources has been emphasized in international studies as a key factor for improving access to anesthesiologists. All eighteen political factors are presented in Fig. 2 of the manuscript.

#### Social area

Social factors include demographic trends, changes in the age pyramid, gender issues, and any stable or emerging phenomenon related to the functioning of society. In this area, 24 factors were mentioned. The most common in this area was adequate opportunities for professional development and assessment of competence. The least mentioned ones were women's empowerment. International studies and low-income countries have identified brain drain and migration as major challenges. In all countries, issues such as job burnout, incompatibility of personal life with work, professional relationships, clinical autonomy, and referrals by other colleagues, especially surgical staff, have been considered. Familiarity with the field has also been raised as a global challenge. Maternity leave has been considered in studies of high-income countries and international research. Adequate opportunities for professional development and assessment of competence have been a matter that has received special attention in all studies, especially at the international level. The rate of retirement and death, population growth or life expectancy, age and sex composition of the population and specialists, and demand for medical, surgical, and acute care have been considered more in high-income countries. Social responsibility was mentioned in studies of low-income countries and job satisfaction in high-income and low-income countries.

#### Economic area

Economic factors are external Trends that affect the economy's performance, which can affect businesses and individuals. In this area, 13 factors were mentioned. The most frequent ones were financial incentives and Workload. The least mentioned was the how to finance. The economic status of the countries has been analyzed more in international studies and high-income countries. Financial incentives and workload are common issues in all countries and have been studied, particularly in high-income countries. The number of graduations has been emphasized more in high-income countries. Attractiveness of the field of anesthesia has been proposed in all countries as one of the key factors for attracting and retaining human resources. Access to facilities, equipment, and medicine was a common issue in all countries, particularly low-income countries.

#### Technological area

Technological factors are external Trends related to the type of technology and its availability and development. Eight factors were identified in this area. The most frequent was medical training opportunities. The least mentioned was efficient referral systems. Medical training opportunities were emphasized in both high-income and low-income countries. Medical and technological advances and distance education were mainly highlighted in international studies as strategies to improve access to anesthesiologists. In all countries, robust information systems for planning were mentioned for proper planning.

#### Environmental area

In this study, five environmental factors were identified. The most common factors was equitable geographic distribution of resources and physicians. The time spent between residence and work (urban/rural), stable ecosystem, place of medical education, and road facilities were mentioned too. Across all countries, equitable geographic distribution of resources and physicians was mentioned as one of the most important challenges and goals. In low-income countries, time spent between places of residence and work (urban/rural) was more prominently examined.

Thus, low-income countries face structural challenges and resource constraints, while high-income countries focus on performance issues and workplace quality, such as retaining motivated professionals. These differences indicate the need for focused and differentiated strategies to address the problems in each group of countries.

## Discussion

In this review, 63 articles published between 2015 and 2023 were examined to identify factors affecting the availability of anesthesiologists using the STEEP Analysis. Also, key disparities between low and high-income countries, underscoring the need for tailored interventions, were identified. Finally, 68 factors were identified in five areas. The high frequency factors were related to the social area, followed by the political area, and the least number of factors were related to the environmental area. The most frequent factors that can signify regional and global concerns about this issue require more attention. The particular focus on task sharing in the political sector suggests that countries need to revise their policies, particularly those related to insurance payments, to address the shortage of anesthesiologists. In countries like Iran, insurance policies reimburse anesthesia services only if delivered by physician anesthesiologists, limiting task-sharing models. Revising these policies to support supervised services by trained non-physician providers could improve access in underserved areas. This strategy aims to enhance anesthesiologists' capacity through team-based care without diminishing their central role. The factors identified in the economic area were the most repeated in the articles, which indicates its important role in providing a health workforce.

### Factors affecting the availability of anesthesiologists using the STEEP analysis

The political area has a significant impact on the availability of anesthesiologists. Political stability, sound health policies, and efficient governance prevent labor migration and brain drain. Health policies can attract and retain anesthesiologists by improving working conditions and career opportunities (1). Factors influencing the provision of human resources include the number of graduates and the use of international physicians. Job burnout can be prevented by improving working conditions and building culture or by the support of hospital managers or politicians (31–33). Most studies addressed the availability or shortage of anesthesiologists, which shows the need for proper planning, primarily based on local needs. These affect job satisfaction and prevent early retirement or leaving the organization.

In high-income countries, clearly defined job requirements and formal backing from national and international organizations contribute significantly to the recruitment and retention of anesthesiologists (34, 35). In contrast, low-income countries often rely on task sharing to address workforce shortages, primarily due to limited resources and weak health system infrastructure (36–38). Global studies emphasize the importance of localized, needs-based planning, particularly when supported by international collaboration. In such contexts, partnerships with international bodies have proven instrumental in improving access to anesthesia services. Overall, in all health systems, the engagement of national and international organizations in developing human resource policies for anesthesia is essential to ensure the sustainability and equitable distribution of services (39, 40).

Studies on social factors showed that this profession is progressing towards feminization. In high-income countries, women's advancement in the anesthesia profession is accompanied by relative support for maternity leave and flexible work schedules, but gender discrimination in hiring practices and pay persists, and maternity leave increases the workload of other colleagues (41–43).

Conversely, while the feminization of the workforce presents challenges, it also offers opportunities for enhancing diversity and improving patient care through varied perspectives and experiences in anesthesiology (44). Social factors may challenge the compatibility of personal life with work. When work expectations and personal life priorities do not align, it can cause stress and dissatisfaction. Research shows that balancing work and personal life through effective time management and managers' support for flexible working arrangements is a key challenge in both groups of countries, but countries with stronger organizational structures manage this balance better (45).

Current shortages in anesthesiology are exacerbated by population growth, increased surgical demand and unwillingness to enter this field of study or drop out (46). Many anesthesiologists, particularly in low-income countries, migrate for better opportunities, further straining local services (24, 44, 47). In some countries, especially low-income countries, anesthesiologists depend on surgeons' decisions in the operating room. This issue can hamper their career progress and even the quality of safe anesthesia management (1, 48, 49).

In both groups of countries, job burnout and work-life conflict have been identified as common barriers that require organizational support and flexible working hours (11, 35, 50–57). Ultimately affecting the workforce's health. In addition, population growth and aging increase the demand for anesthesia services (1, 11, 35, 45, 58–65). Ineffective workforce retention strategies and human resources management reduce the desire for anesthesiologists to work, especially in government organizations and rural areas, leading to immigration or brain drain. Particularly in low-income countries, it increases the desire to migrate to other countries to find job opportunities with better financial incentives. Due to the continuous changes in anesthesia care delivery models, it is necessary to acquire the skills and competencies corresponding to professionalism in anesthesia to meet the changing demands. In all countries, to manage anesthesia safely and continuously improve service quality, there is a need for a set of unique skills that should be included in training programs and continuous professional development, especially for rural areas (26, 34, 35, 62, 66–72). One of the ways for professional development is continuous training, which is challenged due to the need for more trainers or training spaces. Some countries used retired experts and distance education systems to solve this challenge (48, 49, 66, 70, 73–75).

In the economic area of the analysis, the most frequently mentioned factors were financial incentives, access to facilities, equipment, and medicine. The income of this occupational group is low compared to other specialties for various reasons, including not having a private practice. It reduces the desire of students to choose this field of study or to continue working in this specialty. They may become interested in alternative professions due to the low financial incentives and the lack of job satisfaction. With the increased workload, especially among elderly professionals, the probability of medical error increases, and the quality of services decreases (33, 34, 36, 61, 76–83). There are not enough job opportunities for graduates in this field in countries facing economic crises and reduced income in the healthcare system and GDP (36, 61). The demand for specialists can increase when the economy stabilizes, and more people seek to improve their health (33, 70, 73, 84). In addition, managers in unfavorable economic conditions can maintain specialists by improving job characteristics and professional relations (51, 74, 81, 85–87). In high-income countries, fiscal policies have been directed towards maintaining quality, creating incentives, and professional development (23, 35), while low-income countries face resource constraints, dependence on external support, and brain drain (69). This economic gap has a direct impact on health equity and access to anesthesia services and requires structural interventions commensurate with the income level of countries (58).

The technological factors include medical and technological advances (for example, using artificial intelligence in healthcare processes and reducing acute cases by early detection and treatment) that may reduce the need for anesthesiologists. However, it is important to consider that AI should complement human expertise and decision-making processes in healthcare settings, not as a replacement for them. Furthermore, the ethical and legal implications of implementing AI technologies require careful analysis (37, 88). Technology, as a determinant in the distribution and retention of the anesthesia workforce, plays a different role in high-income and low-income countries. In high-income countries, technological advances have not only improved the quality of services but also enabled continuous training, effective resource management, and the implementation of safety protocols. The use of distance learning and advanced information systems has made it possible to access specialized services even in remote areas (37, 73, 89). In contrast, low-income countries face serious challenges in providing basic technology. The lack of appropriate equipment, dependence on foreign aid, and the lack of coherent information systems threaten the sustainability of the workforce. In such circumstances, high workload and lack of technological support lead to burnout and migration of skilled workers (11, 58, 90–92). Therefore, technological policymaking in line with the income level of countries can play a vital role in improving health equity and the efficiency of healthcare systems (36, 89).

Although environmental factors are less discussed in the literature, they are still very important, especially for remote areas with limited access to healthcare facilities and services. Although a common factor across countries in this area is the geographically unequal distribution of resources and physicians, the scale of the challenge varies by income level (66, 75, 93). In high-income countries, the issue is more about policy and job incentives for disadvantaged areas (35), while in low-income countries, the challenges are related to the lack of basic infrastructure such as roads, transportation, and proximity to work (58, 66). This difference suggests that addressing environmental barriers requires local solutions tailored to each country’s economic and geographical circumstances (63).

In a limited number of studies, factors such as efficient referral systems (36), war (54), social accountability (86), insurance laws (94), duty system (94), Stability and political freedom (47, 95), social justice (64), women's empowerment (64), obligations after graduation (24), tax (85), legal issues, and complaints (55) were mentioned. By identifying society's needs and health problems, planning and implementing programs, and evaluating the impact of the programs in solving society's problems (96), governments increase society's satisfaction and access to healthcare services. Implementing an effective referral system can decrease the workload. In regions such as East Africa, where referral networks are fragmented and tertiary hospitals are overcrowded, strengthening referral pathways can help alleviate the excessive workload on anesthesia professionals (36, 97). Legal and regulatory frameworks, tax policies, and insurance laws can create barriers to attracting and retaining anesthesiologists. Drawing on international experiences, particularly in countries where advanced practice nurse anesthetists provide high-quality anesthesia care, revisiting national insurance and regulatory policies may offer a viable strategy to address anesthesiologist shortages, provided that clear standards, oversight, and patient safety safeguards are established (98, 99). In areas facing war or political unrest, health workers may migrate to safer areas and countries, exacerbating the local labor shortage crisis (54).

### Interrelationships between STEEP categories

Although the social, economic, political, technological, and environmental areas and internal components are interrelated, some areas significantly affect others, and not paying attention to them causes challenges (100).

Regarding these connections, we can say that factors such as population growth and migration increase the supply of labor and affect employment opportunities and economic growth. Job dissatisfaction significantly leads to burnout and affects retention and overall economic stability. The lack of gender differences and women's empowerment effectively shape and promote labor force participation policies. Monitoring mechanisms can increase job satisfaction and equity in the workplace and foster a more inclusive environment. The use of technology – such as distance learning platforms – not only facilitates professional development and skills assessment for anesthesia providers but also helps improve referral systems. In regions such as East Africa, where referral networks are fragmented and tertiary hospitals are overcrowded, technology can help reduce unnecessary burden on anesthesia professionals by improving patient referral pathways (93, 101). Promoting innovative technologies in the workplace, such as distance learning and AI-based healthcare solutions, can improve efficiency and job satisfaction, especially in rural and disadvantaged areas. Artificial intelligence (AI) systems can help reduce anesthesiologists’ workload and alleviate cognitive fatigue by enabling automated monitoring of vital signs, providing clinical decision support, and predicting interventions in real time. In rural and remote areas, these technologies can assist non-physician anesthesia providers (NPAPs) by offering immediate clinical guidance and integrating patient information from diverse sources, thereby enhancing the accuracy of decision-making and improving workflow efficiency. However, their design and deployment require careful attention to ethical issues (98). Distance learning reduces costs and expands access to skill development. Strong scheduling systems increase job satisfaction by optimizing the distribution of workload, which is crucial for maintaining employee morale and productivity. Access to urban facilities and resources affects work-life balance. Geographical inequalities can lead to unequal access to opportunities and affect job satisfaction and retention. Effective governance can lead to improved planning and allocation of resources and address environmental challenges in health care. Urbanization affects resource allocation and leads to the unequitable distribution of healthcare professionals in underserved areas [102–105] (106). Robust information systems increase job satisfaction by optimizing workload distribution, which is crucial for maintaining staff morale and productivity. Such systems, including spatial data, are essential for assessing access to health care and planning interventions in underserved areas (58). Task-sharing frameworks increase professional satisfaction by clarifying roles and responsibilities, leading to better workforce engagement (37).

### Limitations and strengths of the study

This scoping review has some inherent limitations. It aimed to map the breadth of global literature rather than evaluate the depth or quality of each included study. Moreover, only English-language publications were included, which may have led to the exclusion of relevant studies in other languages. While the findings offer valuable insights into anesthesia workforce availability, they should be interpreted with caution and within the context of local health system structures and policies.

## Conclusion

Key findings suggest that while high-income countries tend to focus on performance optimization and workplace quality, low-income countries are challenged by structural constraints such as inadequate resources, migration, and poor infrastructure.

Policy Recommendations:

1. High-Income Countries: Focus on improving working conditions, professional development opportunities, and retention strategies, such as job satisfaction and financial incentives. Additionally, leveraging technological advancements, such as AI-based healthcare solutions and distance learning, can alleviate workload pressures, particularly in rural and underserved regions.
2. Low-Income Countries: Address the critical shortage of anesthesiologists by improving the equitable distribution of healthcare resources, enhancing training opportunities, and reducing migration through targeted financial incentives. Developing task-sharing systems and fostering international collaborations can help mitigate workforce shortages. Governments should invest in infrastructure, including road facilities and robust information systems, to improve access to healthcare in remote areas.
3. International Level: A collaborative approach between international organizations, governments, and healthcare institutions can help create frameworks for task-sharing, knowledge exchange, and equitable resource distribution. Policy frameworks should be flexible and adaptable to the unique needs of different regions, ensuring that workforce strategies address local realities while aligning with global standards.

By recognizing the interconnected nature of these factors and implementing region-specific strategies, governments can optimize workforce planning and address the ongoing challenges related to the availability of anesthesiologists. This study highlights the need for tailoring interventions to ensure sustainable anesthesia care across diverse healthcare settings and provides evidence of factors that may be useful in future policymaking.

## Supplementary Information

Supplementary Material 1.

## Data Availability

No datasets were generated or analysed during the current study.

## Abbreviations

SAO: Surgeons, anesthesiologists, and obstetricians
STEEP: Political Social Economic Technological Environmental
