# Supplementary material for: Factors influencing the availability of anesthesiologists: a scoping review
Source: Hum Resour Health. 2025 Oct 24;23:56. doi: 10.1186/s12960-025-01021-3 (PMC12553181; doi:10.1186/s12960-025-01021-3)
Supplement: Supplementary file 1 — Supplementary Material 1. [file 12960_2025_1021_MOESM1_ESM.docx]

Table 1: Characteristics of the reviewed studies

|  | **Author(Year)** | **Type of Study** | **Geographical Scope of the Study** | **factors affecting the availability of anesthesiologists** |
| --- | --- | --- | --- | --- |
| **1** | Cooper, M. G.(2020)(58) | Editorials | Kenya | Equitable geographic distribution of resources and physicians. |
| **2** | Methangkool,E.(2023)(92) | Review | US | Gender Differences. |
| **3** | Mumbwe, M.C(2020)(32) | cross-sectional | Zambia | Job burnout, access to facilities, equipment, medicine, professional relationships, communication, clinical independence, recognition/referral by other colleagues, especially surgical staff, and agreement between clinical positions in intraoperative decisions and working hours. |
| **4** | Basile, E. R.(2023)(33) | Qualitative | International | Gender differences. |
| **5** | Li, H.(2018)(97) | cross-sectional | China | Job burnout, the age and gender of the specials, workload, the characteristics of hospitals at referral levels. |
| **6** | Lundeg, G.(2018)(75) | Review | Mongolia | The length of the training period, workload, medical training opportunities , immigration, the support of national and international organizations, access to facilities, equipment, the time spent between the places of residence and work (urban/rural),equitable geographic distribution of resources and physicians. |
| **7** | Shaw, A. D.(2023)(66) | Editorial | US | Financial incentives, workload, medical training opportunities. |
| **8** | Enright, A. (2017)(48) | Editorial | Canada | medical training opportunities, the support of national and international organizations, professional relationships, communication, clinical independence, recognition/referral by other colleagues, especially surgical staff, and agreement between clinical positions in intraoperative decisions and working hours, distance education. |
| **9** | Drum, E. T.(2017) (37) | Editorial | Canada | The support of national and international organizations, task sharing, medical and technological advances, distance education. |
| **10** | Emala, C.W. S.(2023)(72) | Review | US | The support of national and international organizations , financial incentives, medical training opportunities, adequate opportunities for professional development and assessment of competence, job characteristics, workload, the length of the training period, the support of the hospital manager or policymakers. |
| **11** | Nwokolo, O. O.(2022)(70) | Review | US | Job burnout ،medical training opportunities ،, adequate opportunities for professional development and assessment of competence, gender differences, the age and gender of the specialist, financial incentives. |
| **12** | Shaefi, S.(2023)(49) | Survey | US | Job characteristics, workload, job burnout, professional relationships, communication, clinical independence, recognition/referral by other colleagues, especially surgical staff, and agreement between clinical positions in intraoperative decisions and working hours, incompatibility of personal life with work, medical training opportunities, access to facilities, equipment, medicine. |
| **13** | Meadows, J.W.(2020)(50) | cross-sectional | Bangladesh | Equitable geographic distribution of resources and physicians, access to facilities, equipment, medicine, medical training opportunities, international collaboration on two-way learning about disease patterns, treatment algorithms, research and standards of care, the number of graduations. |
| **14** | Hertzberg, L. .(2021)(73) | cross-sectional | US | Gender differences. |
| **15** | Khan, F. A.  (2022) (100) | cross-sectional | Pakistan | Equitable geographic distribution of resources and physicians, medical training opportunities, adequate opportunities for professional development and assessment of competence, planning based on local needs and resources, medical and technological advances, financial incentives. |
| **16** | Orser, B. A.  (2019)(34) | Review | International | Equitable geographic distribution of resources and physicians, access to facilities, equipment, medicine, financial incentives, adequate opportunities for professional development and assessment of competence, task sharing, medical and technological advances, , workload, distance education, medical training opportunities, the support of national and international organizations, international collaboration on two-way learning about disease patterns, treatment algorithms, research and standards of care, job burnout, the time spent between the places of residence and work (urban/rural). |
| **17** | Asingei, J.  (2023)(74) | cross-sectional | East, Central, and Southern Africa | Equitable geographic distribution of resources and physicians, immigration, workload, medical training opportunities, the support of national and international organizations, adequate opportunities for professional development and assessment of competence, access to facilities, equipment, medicine, financial incentives. |
| **18** | Mayes, L. M.(2018)(67) | cross-sectional | US | Gender differences, the age and gender of the specialists. |
| **19** | Kudsk-Iversen, S. (2018)(11) | Review | International | The use of internationally educated physicians, stability and political freedom, job security, the support of the hospital manager or policymakers, the equitable geographic distribution of resources and physicians, the support of national and international organizations, task sharing, immigration and brain drain, maternity leave, disease burden or epidemics, age and gender characteristics of the population, job characteristics, adequate opportunities for professional development and assessment of competence, incompatibility of personal life with work, employment and job opportunities, financial incentives, workload, job burnout, access to medical training opportunities, access to facilities, equipment, medicine, job satisfaction, the economic status of the countries, distance education, international collaboration on two-way learning about disease patterns, treatment algorithms, research and standards of care, medical and technological advances, robust information systems for planning. |
| **20** | Khan, I.A.  (2023) (1) | Editorial | International | The support of the hospital manager or policymakers, equitable geographic distribution of resources and physicians, the support of national and international organizations, task sharing, demand for medical, surgical and acute care, job characteristics, adequate opportunities for professional development and assessment of competence, financial incentives, workload, alternative professional activities and willingness to work in the private sector, job burnout, access to medical training opportunities, job satisfaction, the economic status of the countries, tuition fees for educational courses . |
| **21** | Davies, J.I. (2018)(23) | cross-sectional | International | The number of graduations, task sharing, familiarity with the field, and attraction to the field. |
| **22** | El Vilaly, M.A.  (2021)(105) | cross-sectional | Nigeria | University acceptance rate, equitable geographic distribution of resources and physicians, population growth or life expectancy, age and gender characteristics of the population, access to city facilities, incompatibility of personal life with work, financial incentives, workload, access to facilities, equipment, medicine, robust information systems for planning, road facilities. |
| **23** | Haller, G.  (2021) (47) | cross-sectional | Switzerland | University acceptance rate, the number of graduations, the use of internationally educated physicians, the length of the training period, employment types, job requirements(standard and pattern of activity), the characteristics of hospitals at referral levels, equitable geographic distribution of resources and physicians, protocols, administrative responsibility of specialists, task sharing, immigration and brain drain, the age and gender of the specialist, gender differences, the rate of retirement and death, maternity leave, disease burden or epidemics, population growth or life expectancy, age and gender characteristics of the population, demand for medical, surgical and acute care, familiarity with the field, adequate opportunities for professional development and assessment of competence, incompatibility of personal life with work, financial incentives, workload, alternative professional activities and willingness to work in the private sector, access to medical training opportunities, attraction with the field, access to facilities, equipment, medicine, medical and technological advance, the variety of anesthesia services. |
| **24** | Department of Health and Aged Care (2015)(35) | Report | Australia | University acceptance rate, the length of the training period, job requirements(standard and pattern of activity), equitable geographic distribution of resources and physicians, task sharing, the age and gender of the specialist, gender differences, maternity leave, population growth or life expectancy, age and gender characteristics of the population, demand for medical, surgical and acute care, leaving or changing jobs, incompatibility of personal life with work, employment and job opportunities, financial incentives, access to medical training opportunities, access to facilities, equipment, medicine. |
| **25** | Muffly, M.K.(2018) (59) | Longitudinal Analysis | The U.S. | The number of graduations, job requirements(standard and pattern of activity), the equitable geographic distribution of resources and physicians, the age and gender of the specialist, the rate of retirement and death, population growth or life expectancy, demand for medical, surgical and acute care, job characteristics, familiarity with the field, employment and job opportunities, alternative professional activities and willingness to work in the private sector, attraction with the field, tuition fees for educational courses. |
| **26** | Zhou, Y.2021)(60) | cross-sectional | The U.S. | The number of graduations, employment types, the characteristics of hospitals at referral levels, the equitable geographic distribution of resources and physicians, regulatory mechanisms and licensing, regulations, how to financing, the administrative responsibility of specialists, task sharing, the age and gender of the specialist, gender differences, the rate of retirement and death, maternity leave, age and gender characteristics of the population, demand for medical, surgical and acute care, access city facilities,  employment and job opportunities, financial incentives, access to facilities, equipment, medicine, the share of the health system budget from the gross national product, the economic status of the countries, robust information systems for planning. |
| **27** | Muffly, M.K.(2016)(61) | cross-sectional | The U.S. | The number of graduations, employment types, the equitable geographic distribution of resources and physicians, obligations after the training period, the age and gender of the specialist, the rate of retirement and death, maternity leave, incompatibility of personal life with the workplace. |
| **28** | Meara, J.G.(2015)(24) | Review | International | The number of graduations, planning based on local needs and resources, activity in two places, equitable geographic distribution of resources and physicians, regulatory mechanisms and licensing, protocols, the support of national and international organizations, task sharing, change of disease pattern, immigration and brain drain, population growth or life expectancy, age and gender characteristics of the population, professional relationships, communication, clinical independence, recognition/referral by other colleagues, especially surgical staff, and agreement between clinical positions in intraoperative decisions and working hours, familiarity with the field, adequate opportunities for professional development and assessment of competence, leaving or changing jobs, workload, attraction with the field, access to facilities, equipment, medicine, international collaboration on two-way learning about disease patterns, treatment algorithms, research and standards of care, medical and technological advances, robust information systems for planning, the time spent between the places of residence and work (urban/rural), sustainable ecosystem, tuition fees for educational courses. |
| **29** | Cooper, M.G.(2016)(62) | Editorial | the Pacific region | The number of graduations, planning based on local needs and resources, task sharing, the rate of retirement and death, age and gender characteristics of the population, professional relationships, communication, clinical independence, recognition/referral by other colleagues, especially surgical staff, and agreement between clinical positions in intraoperative decisions and working hours, job characteristics, workload, job satisfaction |
| **30** | Simkin, S. (2023)(76) | cross-sectional | Canada | The number of graduations, job requirements (standard and pattern of activity), equitable geographic distribution of resources and physicians, immigration, the age and gender of the specialist, the rate of retirement and death, leaving or changing jobs, incompatibility of personal life with work, workload. |
| **31** | Orser, B.A.  (2020)(77) | Review | Canada | The use of internationally educated physicians, job requirements(standard and pattern of activity), planning based on local needs and resources, regulatory mechanisms and licensing, social accountability, the age and gender of the specialist, maternity leave, job characteristics, access to city facilities, adequate opportunities for professional development and assessment of competence, incompatibility of personal life with work, financial incentives, distance education. |
| **32** | Simkin, S.(2023)(68) | Longitudinal Analysis | Canada | The use of internationally educated physicians, the equitable geographic distribution of resources and physicians, the age and gender of the specialist, the rate of retirement and death, disease burden or epidemics, population growth or life expectancy, demand for medical, surgical and acute care, leaving or changing jobs, financial incentives, workload, job burnout, robust information systems for planning, the variety of anesthesia services, place of medical education. |
| **33** | Ulisubisya, M. (2016)(63) | Review | Tanzania | The use of internationally educated physicians, the time spent between the places of residence and work (urban/rural). |
| **34** | Chan, D.M. (2016)(78) | Mixed methods | Rwanda | The use of internationally educated physicians, task sharing, maternity leave, incompatibility of personal life with work, employment and job opportunities, financial incentives, workload, access to facilities, equipment, medicine, and job burnout. |
| **35** | Lyon, C.B.(2016) (51) | Qualitative | Mozambique | The length of the training period, activity in two places, equitable geographic distribution of resources and physicians, obligations after the training period, task sharing, maternity leave, familiarity with the field, adequate opportunities for professional development and assessment of competence, incompatibility of personal life with work, financial incentives, attraction with the field, access to facilities, equipment, medicine, international collaboration on two-way learning about disease patterns, treatment algorithms, research and standards of care, the time spent between the places of residence and work (urban/rural), tuition fees for educational courses. |
| **36** | Khuwaja, A.(2023)(69) | cross-sectional | Pakistan | Employment types, the characteristics of hospitals at referral levels, the equitable geographic distribution of resources and physicians, task sharing, workload, job burnout, and robust information systems for planning. |
| **37** | Hewitt-Smith, A. (2018)(89) | Mixed methods | Uganda | Employment types, brain drain, professional relationships, communication, clinical independence, recognition/referral by other colleagues, especially surgical staff, agreement between clinical positions in intraoperative decisions and working hours, job characteristics, familiarity with the field, adequate opportunities for professional development and assessment of competence, financial incentives, workload, attraction with the field. |
| **38** | Carey, C.(2018)(45) | Review | International | Job requirements(standard and pattern of activity), regulations, task sharing, immigration and brain drain, the age and gender of the specialist, the rate of retirement and death, disease burden or epidemics, age and gender characteristics of the population, demand for medical, surgical and acute care, employment and job opportunities medical and technological advances, the variety of anesthesia services. |
| **39** | Davies, M.(2022)(83) | Guidelines | International | Planning based on local needs and resources, regulations, maternity leave, disease burden or epidemics, community health status, job characteristics, incompatibility of personal life with work, financial incentives, workload, job burnout, and job satisfaction. |
| **40** | Yang, L.(2017)(52) | Survey | China | Planning based on local needs and resources, activity in two places, professional relationships, communication, clinical independence, recognition/referral by other colleagues, especially surgical staff, an agreement between clinical positions in intraoperative decisions and working hours, adequate opportunities for professional development and assessment of competence, workload, the economic status of the countries. |
| **41** | Law, T.(2019)(86) | Review | International | Planning based on local needs and resources, regulatory mechanisms and licensing, adequate opportunities for professional development and assessment of competence, employment and job opportunities, workload, access to facilities, equipment, medicine, distance education, and robust information systems for planning. |
| **42** | Epiu, I. (2017)(36) | cross-sectional | East Africa | Planning based on local needs and resources, equitable geographic distribution of resources and physicians, protocols, task sharing, familiarity with the field, financial incentives, workload, alternative professional activities and willingness to work in the private sector, attraction with the field, access to facilities, equipment, medicine, the share of the health system budget from the gross national product, efficient referral systems. |
| **43** | Brouillette, M.A.  (2017)(71) | Observational | Ghana | Stability and political freedom, immigration, job characteristics, familiarity with the field, financial incentives, and attraction to the field. |
| **44** | Khan, F.A.  (2018)(94) | Review | International | Job security, the support of the hospital manager or policymakers, the support of national and international organizations, task sharing, immigration and brain drain, women's empowerment, population growth or life expectancy, demand for medical, surgical and acute care, familiarity with the field, adequate opportunities for professional development and assessment of competence, security and social justice, employment and job opportunities, attraction with the field, financial incentives, access to facilities, equipment, medicine, the share of the health system budget from the gross national product, distance education, sustainable ecosystem, tuition fees for educational courses. |
| **45** | Hinkelmann, J.(2018)(64) | Review | German | The characteristics of hospitals at referral levels, the age and gender of the specialist, gender differences, maternity leave, demand for medical, surgical and acute care, professional relationships, communication, clinical independence, recognition/referral by other colleagues, especially surgical staff, and agreement between clinical positions in intraoperative decisions and working hours, incompatibility of personal life with work. |
| **46** | Rama-Maceiras, P. (2015)(43) | Review | International | The support of the hospital manager or policymakers, maternity leave, professional relationships, communication, clinical independence, recognition/referral by other colleagues, especially surgical staff, and agreement between clinical positions in intraoperative decisions and working hours, job characteristics, leaving or changing jobs, incompatibility of personal life with work, job burnout. |
| **47** | Dohlman, L.E.  (2017)(53) | Review | International | The support of the hospital manager or policymakers, equitable geographic distribution of resources and physicians, regulations, the support of national and international organizations, how to financing, task sharing, change of disease pattern, immigration, gender differences, maternity leave, community health status, professional relationships, communication, clinical independence, recognition/referral by other colleagues, especially surgical staff, and agreement between clinical positions in intraoperative decisions and working hours, job characteristics, familiarity with the field, adequate opportunities for professional development and assessment of competence, incompatibility of personal life with work, employment and job opportunities, financial incentives, attraction with the field, access to facilities, equipment, medicine, job satisfaction, war and sanctions, tuition fees for educational courses. |
| **48** | Sousa, A.R.C. (2018)(54) | literature review | International | The support of the hospital manager or policymakers, equitable geographic distribution of resources and physicians, legal issues and complaints, the administrative responsibility of specialists, immigration, gender differences, the rate of retirement and death, demand for medical, surgical and acute care, professional relationships, communication, clinical independence, recognition/referral by other colleagues, especially surgical staff, and agreement between clinical positions in intraoperative decisions and working hours, job characteristics, adequate opportunities for professional development and assessment of competence, incompatibility of personal life with work, security and social justice, financial incentives, workload, job burnout, the economic status of the countries. |
| **49** | Fernandes, N.L.  (2023)(55) | Review | International | Equitable geographic distribution of resources and physicians, task sharing, immigration, financial incentives, and access to facilities, equipment, and medicine. |
| **50** | Merchant, A.(2015)(38) | Survey | Guatemala, Guyana, Laos, Mozambique | Equitable geographic distribution of resources and physicians, task sharing, and access to facilities, equipment, and medicine. |
| **51** | Zhang, C.(2021)(79) | cross-sectional | China | Equitable geographic distribution of resources and physicians, familiarity with the field, employment and job opportunities, financial incentives, workload, job burnout, attraction with the field, access to facilities, equipment, medicine, and job satisfaction. |
| **52** | The Royal College of Anaesthetists: Churchill House (2020)(80) | Report | U.K. | Equitable geographic distribution of resources and physicians, regulations, taxes, the age and gender of the specialist, demand for medical, surgical and acute care, employment and job opportunities, financial incentives, the economic status of the countries, and medical and technological advances. |
| **53** | Zha, Y.T. (2021)(84) | cross-sectional | Guatemala | Protocols, the rate of retirement and death, robust information systems for planning. |
| **54** | Wang, J.-O.(2015)(90) | cross-sectional | Taiwan | duty system, insurance laws, maternity leave, professional relationships, communication, clinical independence, recognition/referral by other colleagues, especially surgical staff, agreement between clinical positions in intraoperative decisions and working hours, incompatibility of personal life with work, financial incentives, workload, and job satisfaction. |
| **55** | Baron, E.L.(2020)(93) | Review | International | The rate of retirement and death, maternity leave, professional relationships, communication, clinical independence, recognition/referral by other colleagues, especially surgical staff, an agreement between clinical positions in intraoperative decisions and working hours, adequate opportunities for professional development and assessment of competence, leaving or changing jobs, incompatibility of personal life with work, job burnout. |
| **56** | Gajewski, J.(2020)(56) | Mixed methods | Malawi, Tanzania, and Zambia | Task sharing, social accountability, maternity leave, employment and job opportunities, financial incentives, workload, job burnout. |
| **57** | Baird, M.(2015)(85) | Survey | The U.S. | Task sharing, the age and gender of the specialist, gender differences, the rate of retirement and death, age and gender characteristics of the population, demand for medical, surgical and acute care, and the economic status of the countries. |
| **58** | Baxter, L.S(2017)(65) | Observational | Madagascar | Access city facilities, access to facilities, equipment, medicine, task sharing. |
| **59** | Bouchard, M.E. (2020)(81) | Cross-sectional | Uganda, Sierra Leone | Financial incentives, workload, task sharing, robust information systems for planning. |
| **60** | Federspiel, F. (2015)(91) | Mixed methods | International | Task sharing, international collaboration on two-way learning about disease patterns, treatment algorithms, research and standards of care. |
| **61** | Skelton, T(2020)(40) | Qualitative | Rwanda | The time spent between the places of residence and work (urban/rural), workload, job requirements(standard and pattern of activity), medical training opportunities, the support of national and international organizations, job satisfaction, familiarity with the field, adequate opportunities for professional development and assessment of competence, social accountability, immigration, brain drain, leaving or changing jobs, financial incentives, professional relationships, communication, clinical independence, recognition/referral by other colleagues, especially surgical staff, and agreement between clinical positions in intraoperative decisions and working hours, activity in two places, the support of the hospital manager or policymakers, incompatibility of personal life with work, access to facilities, equipment, medicine, job burnout, attraction the field. |
| **62** | Law, T. (2021)(12) | Survey | Uganda | Job satisfaction, the time spent between the places of residence and work (urban/rural), social accountability, job characteristics, medical training opportunities, access to city facilities, adequate opportunities for professional development and assessment of competence, financial incentives, workload, incompatibility of personal life with work, access to facilities, equipment, medicine, activity in two places, alternative professional activities and willingness to work in the private sector. |
| **63** | Cooper, A. E.(2017)(57) | Review | U.K. | Demand for medical, surgical, and acute care, job burnout, workload, equitable geographic distribution of resources and physicians, international collaboration, medical and technological advances, task sharing, adequate opportunities for professional development and assessment of competence, support of national and international organizations, professional relationships, financial incentives, medical training opportunities, security and social justice |
